# A Modeling Insight into Adipose-Derived Stem Cell Myogenesis

**DOI:** 10.1371/journal.pone.0137918

**Published:** 2015-09-17

**Authors:** Rajiv S. Deshpande, Warren L. Grayson, Alexander A. Spector

**Affiliations:** Department of Biomedical Engineering, Johns Hopkins University, Baltimore, Maryland, United States of America; University of California, San Diego, UNITED STATES

## Abstract

Adipose-derived stem cells (ASCs) are clinically important in regenerative medicine as they are relatively easy to obtain, are characterized by low morbidity, and can differentiate into myogenic progenitor cells. Although studies have elucidated the principal markers, PAX7, Desmin, MyoD, and MHC, the underlying mechanisms are not completely understood. This motivates the application of computational methods to facilitate greater understanding of such processes. In the following, we present a multi-stage kinetic model comprising a system of ordinary differential equations (ODEs). We sought to model ASC differentiation using data from a static culture, where no strain is applied, and a dynamic culture, where 10% strain is applied. The coefficients of the equations have been modulated by those experimental data points. To correctly represent the trajectories, various switches and a feedback factor based on total cell number have been introduced to better represent the biology of ASC differentiation. Furthermore, the model has then been applied to predict ASC fate for strains different from those used in the experimental conditions and for times longer than the duration of the experiment. Analysis of the results reveals unique characteristics of ASC myogenesis under dynamic conditions of the applied strain.

## Introduction

Adipose-derived stem cells (ASCs) obtained from lipoaspirate tissue provide an easily accessible, abundant source for autologous cells and thus have a great potential to tissue engineering and cell therapies [1–3]. ASCs have demonstrated their multi-lineage ability by differentiating into osteogenic, chondrogenic, vascular, neuronal [4, 5, 3, 6] as well as myogenic phenotypes [7]. It has been shown that during myogenesis, ASCs express the same myogenic markers (PAX7/3, Desmin, MyoD, and MHC) and exhibit similar morphological changes (cell alignment and elongation) as satellite cells show in vivo [8, 9]. The satellite cells are stem cells that repair and develop skeletal muscle. Despite such promising properties for myogenic purposes, ASCs demonstrate a relatively low differentiation ability; for example, we have shown that these cells subjected to myogenic medium do not express the late marker, MHC [8]. Therefore, there is a need for an improvement in the ASC myogenic capacity. It has recently been shown that the mechanical and biophysical factors, such as cell shape [10], substrate stiffness [11], and surface topography [12, 13] play important roles in stem cell fate. Moreover, applied loading (strain) has a substantial effect on stem cell myogenesis as the effects of such strain were explored in the differentiation of ASC and mesenchymal stem cells (MSCs) into smooth muscle cells [14, 15]. We have previously considered mechanical cues relevant to the physiological conditions and shown that the application of cyclic uniaxial strains for one hour a day with an amplitude of 10% and frequency of 0.5 Hz can significantly improve ASC myogenesis in vitro [8]. In particular, a significant percent of cells in that study expressed the late myogenic markers, MyoD and MHC, and fused into multinuclear myotubes.

Despite such an outcome, a better understanding and optimization of ASC myogenesis under different conditions are not clear but important. In this regard, a computational model can interpret the experimental observations by using unifying concepts, predict the data beyond the experiment, suggest additional factors to measure, and be extended to study the effects of more complicated culture conditions. Recently, a number of mathematical (computational) models have been developed to predict or explain stem cell fate under different conditions. One approach considers major factors such as a genetic (signaling) network and interprets stem cell differentiation from the mathematical standpoint of the system’s bifurcations, i.e. the appearance of additional steady states when an external parameter reaches a critical value [16, 17]. A physiological model of hematopoietic cell differentiation [18] has shown that differentiation is governed by the value of a complex parameter that characterizes the ligand/receptor signaling. Another method, suited for kinetics studies, treats stem cell differentiation as a transition through several stages described in terms of fluxes of cell number in a given stage to the next one [19]. The latter approach was previously applied to hematopoietic cells and, among other results, revealed the importance of a feedback signal to make the proposed model more representative of experimental observations [19]. Another important application of kinetic models that incorporate various forms of feedback (signaling) was the analysis of cancer cells [20]. If a model includes multiple interconnected factors, the continuous approach using differential equations may not be effective, and the model of component interaction can be reduced to simpler logical (Boolean) variables [17]. Such findings encourage further implementation of computational methods to ASC differentiation.

We have recently developed an experimental and modeling study to describe in-vitro ASC myogenesis [8]. The proposed model [8] interprets ASC myogenesis as a transition through five stages where each of them is determined by a combination of four myogenic markers (PAX7, Desmin, MyoD, and MHC) whose expression is measured in the experiment. Although the approach [8] reproduced important features of the experimental data, we present here a critical extension of the model that a) broadens its biological framework by incorporating the interactions with and feedback from the cellular environment, b) obtains a better quality approximation of the cell number in each state under static and dynamic conditions, and c) predicts the system’s behavior beyond the current experimental conditions, such as for different strains and longer times. Such an advanced model will provide for a better understanding of ASC myogenesis and associated mechanotransduction as well as aid in the design of further experiments to more deeply understand these processes.

**Fig 1:**
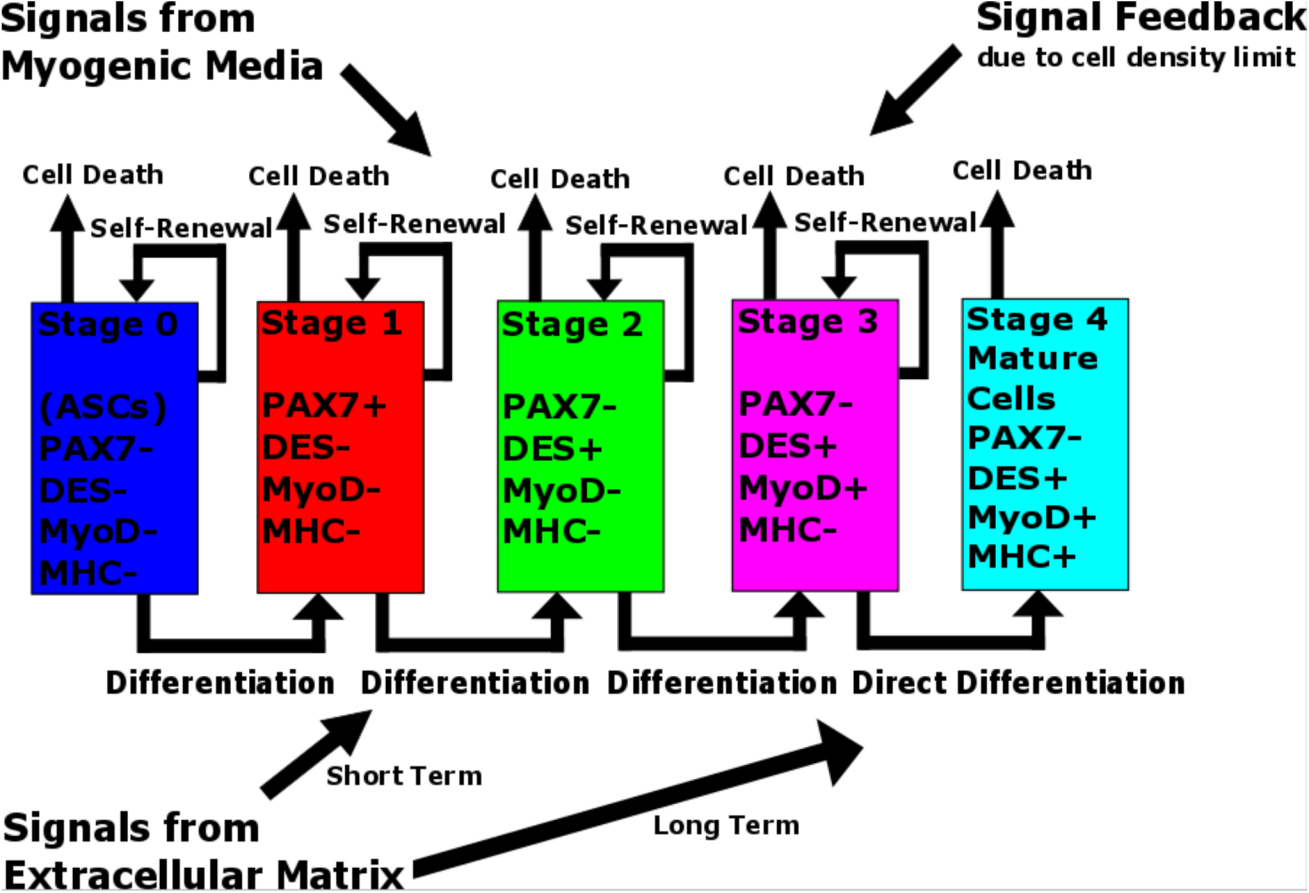
Conceptual model of ASC myogenesis. Five stages of the process are characterized by different combinations of myogenic markers. The cell number in each stage changes with cell proliferation and death. The stage-to-stage progression occurs via asymmetric division or direct differentiation. The process is modulated by signals from the myogenic medium and extracellular matrix (short- and long-term) as well as the feedback associated with a limit in the cell density in the culture.

In the present paper, we propose a nonlinear model that has first been trained by our experimental data of ASC-myogenesis [8] and then implemented to predict the kinetics for different dynamic conditions. Compared to the model version in [8], we redefine the main stages of the kinetics to accurately reflect the role of the factor PAX7 whose expression first increases to activate the original stem cells and then decreases to promote the process of ASC differentiation. In addition, we assume direct differentiation (without division) in the later stages of the process. We furthermore replace the switches previously related [8] to the particular moments of time with a set of more general time-independent conditions expressed in terms the system variables. Such conditions are biologically associated to signals that arise from the interactions with the myogenic medium and extracellular matrix (ECM). Finally, we introduce a feedback signal that is based on cell density thresholds in the culture. The main features of the model proposed here are shown in introductory Fig 1. We adjust the parameters of our advanced model to reproduce the cell number data from the experimental time course at each stage of the process [8]. We then extend our model to intermediate values of strain and obtain the results for times longer than the duration of the experiment. To do this, we use strain-linear approximations of the model parameters corresponding to 10% (dynamic case) and 0% (static case) strain and compute the kinetics for different strains from 1% through 15%. We found that below the critical value of strain (about 2%) the computed kinetics is similar to the static case. On the other hand, for larger strains, the kinetics exhibit a dynamic pattern where the times of appearance of the later stages, 3 and 4, as well as cell numbers in these stages, are strain-dependent.

## Results and Discussion

### Training the Model by Using the Experimental Data

Our extended model is significantly broader than the original version in [8] due to new conditions and parameters included. Thus, we start by adjusting the model parameters using the experimental data from [8, see there also the details of the experiment]. In particular, we use the experimental numbers of cells expressing each of the four myogenic markers (Figure 4c in [8]) as well as the total number of cells (Figure 2b in [8]) reported on days 1, 3, 7, 14, and 21 under static and dynamic (cyclic strain of amplitude 10% and 0.5 Hz frequency) conditions.

Based on the definition of the cell stages in our model (Fig 1), the numbers, n_1_, n_2_, n_3_ and n_4_, of cells in stages 1, 2, 3, and 4, respectively, are related to the experimental values n_PAX7_, n_Des_, n_MyoD_, and n_MHC_ expressing PAX7, Desmin, MyoD, and MHC, respectively, as follows:

$$n_{1}=n_{PAX7}$$ (1)

$$n_{2}=n_{Des}-n_{MyoD}$$ (2)

$$n_{3}=n_{MyoD}-n_{MHC}$$ (3)

$$n_{4}=n_{MHC}$$ (4)

**Fig 2:**
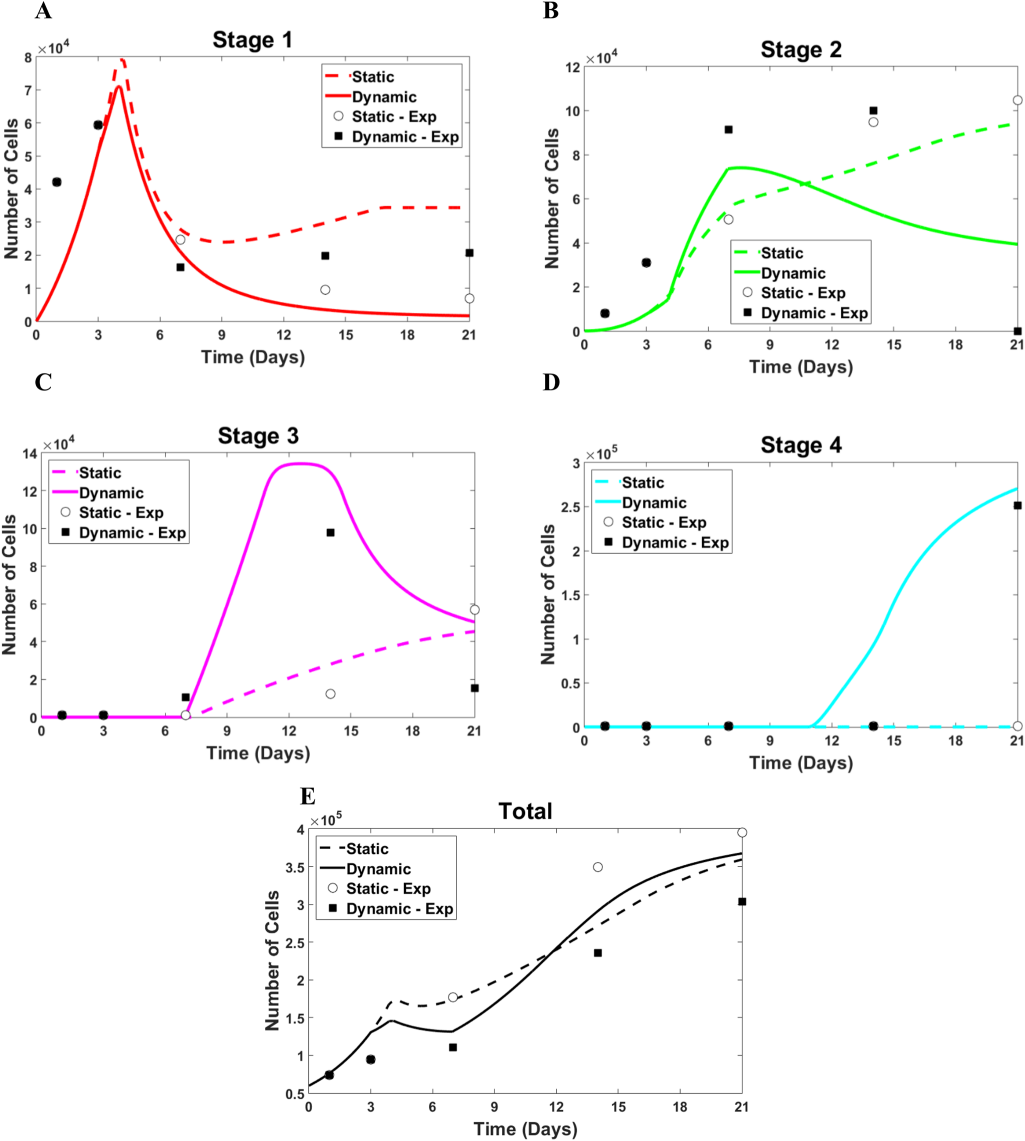
The results from the model with adjusted parameters vs. experimental data. The static case: dashed line (model) and open circles (experiment), and the dynamic case: solid line (model) and filled squares (experiment). (A) Cell number in stage 1, (B) cell number in stage 2, (C) cell number in stage 3, (D) cell number in stage 4, and (E) total number of cells.

These relations are broader than those in [8] because they also include the factor PAX7. We determine the left-hand side values in Eqs 1–4 from the experiment and show the results in Fig 2 as open circles (static case) and filled squares (dynamic case). The number of stem cells expressing none of the four factors is not directly reported in the experiment but rather are included in the total cell number, n_tot_. The modeling data for n_1_, n_2_, n_3_, n_4_, and n_tot_ are presented in Fig 2 via dashed (static cases) and solid (dynamic cases) lines.

**Fig 3:**
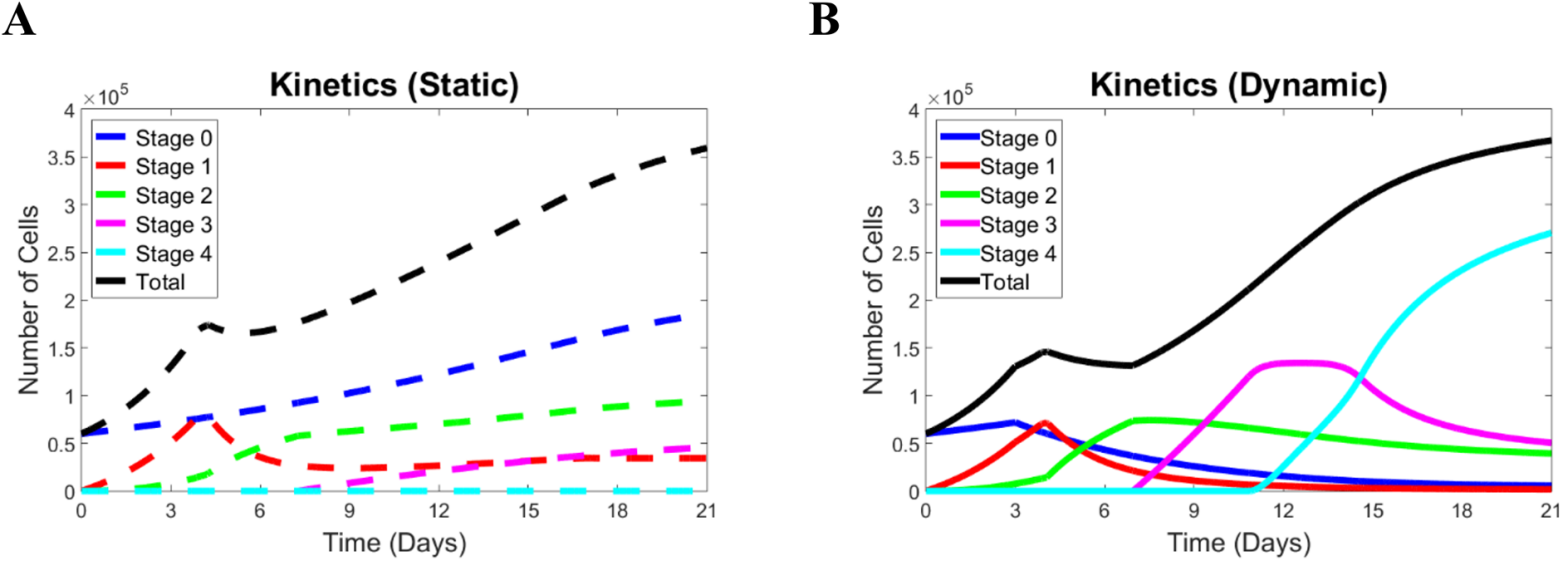
Stage kinetics from the model with adjusted parameters. (A) Dashed lines represent the static case, (B) Solid lines represent the dynamic case.

After adjusting the model parameters, the results reproduce the major features of the kinetics observed in the experimental data. Indeed, the computed n_1_ number undergoes a sharp increase, reaches a maximum at around day 3 of the experiment, and then drops afterwards (Fig 2A). This behavior is typical of both static (open circles) and dynamic (filled squares) cases. The consistent overlap of circles and squares only prior to day 3 indicates that the ASC cultures take different trajectories when strain is applied. Also, the number of cells in the second stage, n_2_, (Fig 2B) monotonically increases throughout the time of the experiment in the static case (dotted line and open circles). In the dynamic case, however, it has a maximum and drops (solid line and filled squares). The number of cells in stage#3, n_3_, stays close to zero until day 7 in the experiment. Similarly, in the model, its deviation from zero is associated with a decreased expression of the factor, PAX7. Previous studies have provided evidence for the mutual inhibition between MyoD and PAX7 [21, 22]. Accordingly, with the release of MyoD, PAX7 levels drop, and the number of cells in stage#3 increases, reaches a maximum, and then decreases due to differentiation in the dynamic case (solid line and filled squares in Fig 2C). In the static case, n_3_ increases after the release of MyoD too but monotonically increases through day 21 of the experiment (solid line and open circles in Fig 2C). Finally, the cells in the fully differentiated stage#4 are completely absent in the static case (Fig 2D). In the dynamic case, however, the terminally differentiated cells start appearing at about day 14 of the experiment, their number, n_4_, continue increasing through the final day 21 of the experiment with a slowing rate closer to the end of the experiment(solid line and filled squares in Fig 2D). Such a change in the rate of n_4_ increase in the dynamic case is an indication of stabilization for a longer time. Such times are not reached in Fig 2 but they are presented in the predicted data corresponding to a broader dynamic case for different strains below. In the current version of the model, the time at which n_4_ deviates from zero depends on whether the factor MyoD (n_3_) reaches a threshold. Such a threshold is reached in the dynamic case of 10% strain but is not reached in the static case as shown by the levels of n_3_ in Fig 2C in these two cases. The rationale for such a criterion for the appearance of terminally differentiated cells can be supported by in vivo [23, 24] and in vitro [8, 9] observations of the final stages of myogenesis. During these stages, myoblasts (in vivo) and precursor cells (in vitro) align, interact, and fuse into myotubes. These processes are likely consequences of the earlier strain application and strain-dependent signaling through the cell-ECM focal adhesions and cell cytoskeleton similar to other adherent cells [25, 26]. It is known that MyoD is not the only indicator of the expression of MHC, and a number of characteristic factors (proteins) are involved on the processes cell-cell contact and fusion during myogenesis [27–30]. Nevertheless, in the experiment with ACS myogenesis [8], the expression of MyoD accompanies cell alignment and fusion, and, for that reason, the MyoD factor has been chosen to represent the precursor compound for the expression of the latest marker MHC. Fig 2E presents the kinetics of the total number of cells in the static (open circles and dashed line) and dynamic (filled squares and solid line) cases, respectively. In both cases, n_tot_ monotonically increases with a slowing rate near the end of the experimental time period. This slowing-down is more pronounced in the dynamic case and is a result of feedback signal limiting the growth in cell numbers when the cell density (total cell number) has reached the critical level. In the static case, such a threshold has not been reached before day 21, but the feedback will have its effect at later times as it is shown below for extended periods of time. The holistic kinetics pictures, including all stages and total numbers of cells, are summarized in Fig 3A and 3B for the static and dynamic cases, respectively.

### Static and Dynamic Patterns of Kinetics

The kinetics of cells in the five stages (i = 0,1,2,3,4) are governed by the proliferation, p_i_, death, d_i_, and direct differentiation (in the later stages) rates, D_i_, as well as self-renewal rates, r_i_. The values of these parameters resulting from the model adjustment to the experimental data are presented in Table 1 (grayed out entries indicate that such parameters are not used). The parameters remain constant and change only at the logical switches as determined by the external signals. In the table, four such switches (absent in the earlier version of the model in [8]) are specified: the moment at which the n_1_-threshold determined by a signal from the myogenic medium (both in the static and dynamic cases); the moment of strain application that triggers the changes in the initial kinetics in the dynamic case governed by the early cell/ECM interaction; the moment when n_1_ drops below a threshold that initiates the release of MyoD (both in the static and dynamic cases), and, finally, the time when n_3_ reaches a threshold that governs the release of MHC (which is included in both static and dynamic cases but actually implemented only in the dynamic case.

**Table 1:** Parameters of the computational model in the static and dynamic cases.

Day 0 (myogenic medium is applied) through day 3 (strain is applied)					
Stage	0	1	2	3	4
r_static	0.8	0.9	1.0	Not Involved (n.i.)	
p_static	0.4	0.6	0.2	n.i.	
d_static	0.2	0.2	0.2	n.i.	
r_dynamic	0.8	0.9	1.0	n.i.	
p_dynamic	0.4	0.6	0.2	n.i.	
d_dynamic	0.2	0.2	0.2	n.i.	
Day 3 until n_1_-upper threshold is met					
Stage	0	1	2	3	4
r_static	0.8	0.9	1.0	n.i.	
p_static	0.4	0.6	0.2	n.i.	
d_static	0.2	0.2	0.2	n.i.	
r_dynamic	0.6	0.9	1.0	n.i.	
p_dynamic	0.1	0.3	0.4	n.i.	
d_dynamic	0.2	0.0	0.1	n.i.	
Upper n_1_-threshold is met through lower n_1_-threshold is met					
Stage	0	1	2	3	4
r_static	0.8	0.0	1.0	n.i.	
p_static	0.4	0.1	0.2	n.i.	
d_static	0.2	0.6	0.2	n.i.	
r_dynamic	0.6	0.0	1.0	n.i.	
p_dynamic	0.1	0.2	0.4	n.i.	
d_dynamic	0.2	0.35	0.3	n.i.	
Lower n_1_-threshold is met through upper n_3_-threshold is met					
Stage	0	1	2	3	4
r_static	0.8	0.0	0.8	1.0	n.i.
p_static	0.4	0.1	0.2	0.1	n.i.
d_static	0.2	0.6	0.2	0.25	n.i.
r_dynamic	0.6	0.0	0.5	1.0	n.i.
p_dynamic	0.1	0.2	0.4	0.3	n.i.
d_dynamic	0.2	0.35	0.1	0.20	n.i.
Upper n_3_-threshold is met through time end					
Stage	0	1	2	3	4
r_static	0.8	0.0	0.8	1.0	0.0
p_static	0.4	0.1	0.2	0.1	0.0
d_static	0.2	0.6	0.2	0.25	0.0
D_static	n.i.			0.05	n.i.
r_dynamic	0.6	0.0	0.5	1.0	0.0
p_dynamic	0.1	0.2	0.4	0.3	0.0
d_dynamic	0.2	0.35	0.1	0.20	0.0
D_dynamic	n.i.			0.55	n.i.

The values of the proliferation, p_i_, death, d_i_, rates, self-renewal ratios, r_i_, and direct differentiation rate, D, in five stages of ASC myogenesis under static (no strain) and dynamic (10% strain) conditions.

There are key combinations of the model parameters that determine the pattern of the stage kinetics. In the stages where differentiation occurs via the asymmetric division, such key parameters are as follows:

$$l_{i}=\left(2r_{i}-1\right)p_{i}-d_{i}\;i=0,1,2$$ (5)

For n_3—_kinetics, as r_3_ equals one, such a parameter takes the form:

$$l_{3}=p_{3}-d_{3}-D_{3}$$ (6)

Depending on whether the l_i_-combination is positive or negative, the corresponding n_i_ increases or decreases, respectively. In the beginning, the application of myogenic medium provides a positive l_0_-combination in both the static and dynamic cases; as a result, the original stem cells proliferate and grow in number. This trend is not interrupted in the static case as no strain is applied. Conversely in the dynamic case, the parameters change with strain application and cause l_o_ to switch signs to a negative value, which results in a decrease of the stem cell number, when compared to the static case. The initial increase in the cell number in stage#1 is provided by a significantly positive value of l_1_ with almost complete self-renewal and relatively low death rate. After reaching the n_1_-threshold, both the dynamic and static parameters change to those with low self-renewal and higher death rates, resulting in a significantly negative l_1_ and a further decrease in n_1_.

According to our concept of the appearance of later myogenic markers, cells do not move into stage#3 until MyoD is released when PAX7 expression (n_1_) drops to a minimal level. Thus, the equation for n_3_ is not integrated, and, consistently, the equation for the previous stage, n_2_, does not include the term responsible for the differentiation into stage#3 (Table 1). After the n_1_-threshold is reached, the equations for n_3_ are included in the computations, and the corresponding parameters provide the n_3_-increase, which is more significant in the dynamic case. Similar to the treatment of the MyoD release, the equation for n_4_ is not integrated and the differentiation term is not included in the equation for n_3_ until n_3_ reaches its respective threshold. Since the n_3_-increase is significantly greater in the dynamic case, the conditions of n_4_-release are satisfied at around day 12, after which the number of cells in terminally differentiated stage starts increasing (see more details on the integration of the kinetic equation below).

The main difference between the kinetics of the two cases is that in the static case, the original stem cells continue increasing in number throughout the whole time of the experiment, and the terminally differentiated cells do not appear at all. In contrast, in the dynamic case, the number of stem cells starts decreasing after the strain application, and terminally differentiated cells become dominant toward the end of the experiment, a trend that continues throughout the experiment. Note that the values of the parameters in Table 1 correspond to two particular values, 10% and 0%, of the applied strain. Showing them back-to-back in the table helps to compare the static and dynamic patterns. Importantly, these values are used as the basic points for linear approximations of the model parameters in our study of the strain-dependence of the kinetics of ACS myogenesis.

### Model Predictions for Different Strains

**Fig 4:**
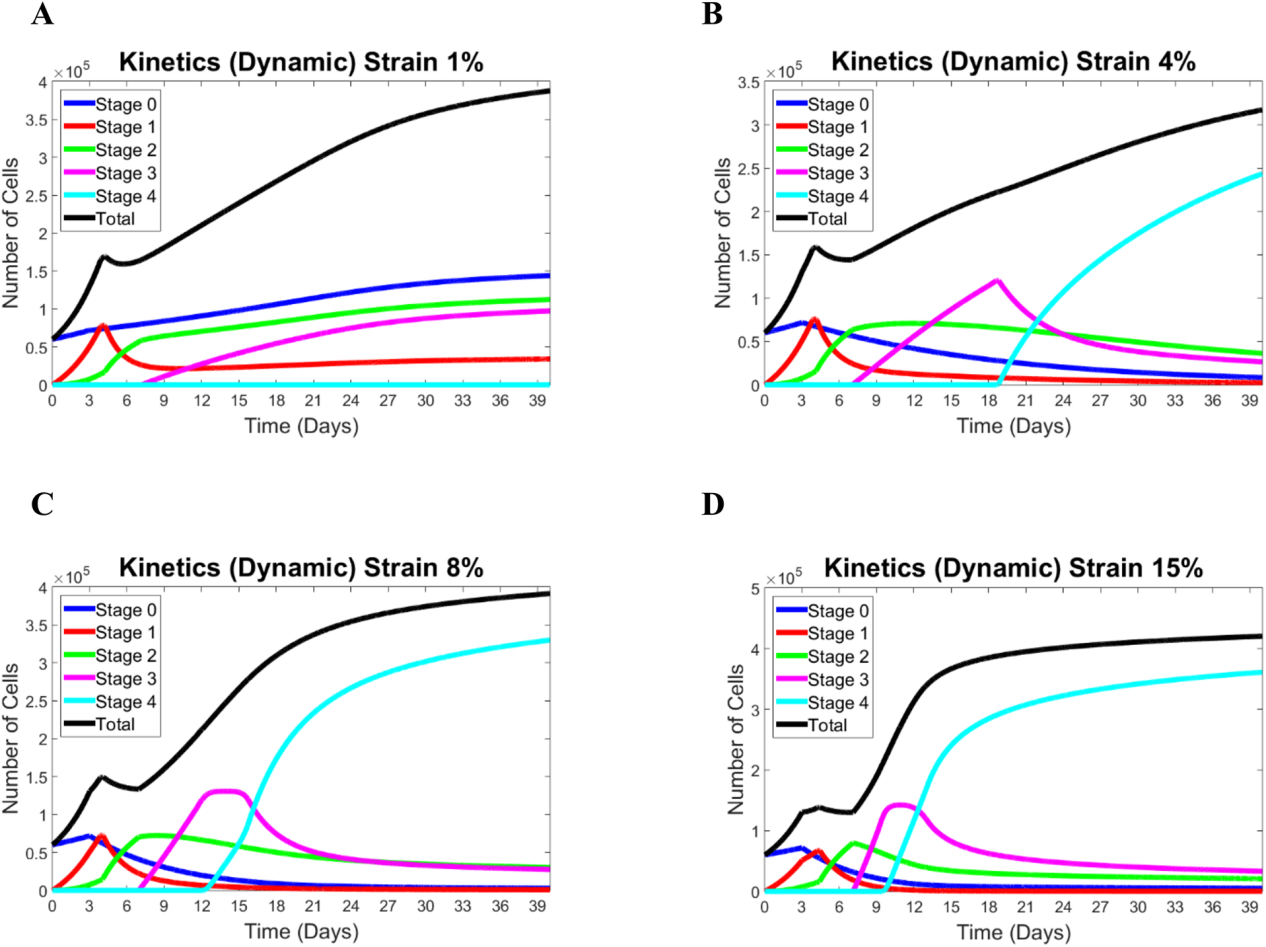
Modeling results for different strains: (A) 1%, (B) 4%, (C) 8%, and (D) 15%. For the low level of strain (1%), the kinetics excibits a staic-like pattern, and for the greater strains (4, 8, and 15%) the pattern of kinetitcs is dynamic-like.

Fig 4 presents the stage kinetics for four different values of the applied strain, 1%, 4%, 8%, and 15% and for an extended time interval of 40 days. These data provide a broader framework to describe two patterns, “static-like” and “dynamic-like”, of stage transition. In the case of a small strain in Fig 4A, the original stem cells increase in number throughout the whole time interval despite a slight change in the parameters at the moment of strain application. Cells expressing PAX7 (n_1_) behave similarly to those in the purely static case considered above. Moreover, cells in the late stage#3 increase in number after their release at about day 7, which is the same as in the static case because the n_1_-decrease is the same. The number of cells in stage#3, however, does not reach the threshold required to release the cells in terminally differentiated stage. Thus, by the end of the considered time interval, the fraction of n_3_ stem cells becomes the largest one, as there are no cells that reach terminally differentiated stage#4. This is qualitatively similar to the pure static case. Further analysis, not presented, shows that a similar pattern remains up to a strain of about 2%, above which it starts changing.

**Fig 5:**
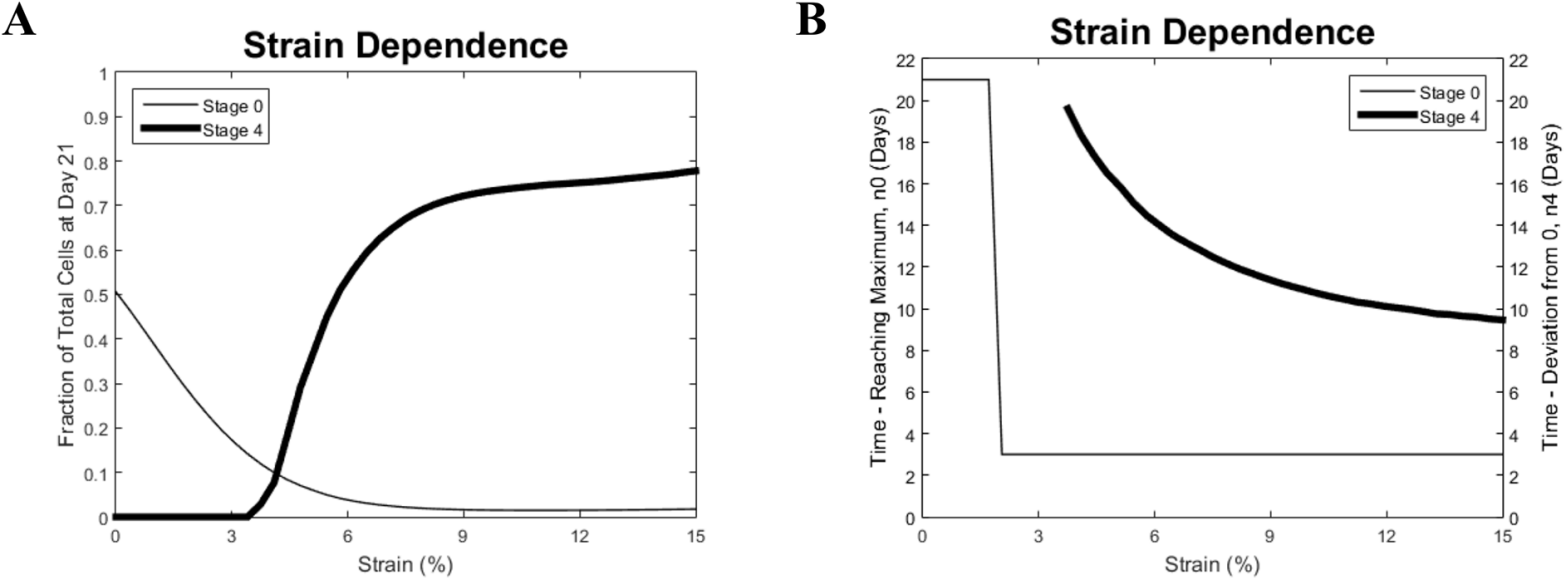
Strain dependence. **Static- and dynamic-like patterns.** (A) Fractions of the total cell number of stem cells (stage#0, thin line)) and terminally differentiated cells (stage#4, thick line) at the end of the experiment (day 21) and (B) time (day) of stem cell number reaching the maximum over the time of the experiment (thin line) and time (day) of the first appearance of terminally differentiated cells (thick line).

In all cases of strains presented in Fig 4B–4D, the number of cells in stage#0 decreases after strain application, the number of cells in stage#3 reaches its threshold to provide the release of cells in the terminally differentiated stage, and the cell number in stage#4 starts monotonically increasing after that. Moreover, in all the cases in Fig 4B–4D, the number of stem cells becomes small while the fraction of terminally differentiated cells becomes dominant. This is consistent with the dynamic pattern of kinetics. However, analysis reveals differences in the kinetics in these three cases which indicate the strain-dependence of important features of the dynamic pattern. First, the increase in n_3_ after its release is steeper, and the moment when n_3_ reaches its threshold occurs earlier for larger strains. Such moments happen on days 10 and 19, respectively, in the cases of strain of 15% and 4%. Second, the number of terminally differentiated cells at the end of the same time period is different and higher for larger strains (3.7x10^5^ and 2.5x10^5^ cells for strains of 15% and 4%, respectively). Also, the rate of increase of n_4_ decreases with time in the case of larger strains (compare the results in Fig 4B and 4D). The number of cells in all stages stabilizes with time if we neglect the death rate in stage 4 (not shown). However, the time required until such stabilization is different and is shorter for larger strains. Thus, the main difference between two patterns is the fraction of the remaining stem cells and that of the cell in the terminally differentiated stage. There is a critical value of the strain when the static pattern transforms into the dynamic one, and the dynamic features accordingly become more pronounced for larger strains. Fig 5 summarizes the difference between static and dynamic kinetics of n_0_ and n_4_ as well as their strain-dependence. Fig 5A shows the strain-dependence of the fractions of stem cells (n_0_/n_tot_) and terminally differentiated cells (n_4_/n_tot_) estimated at day 21 (end of the experiment). In the purely static case, the original stem cells take about 50% of the total population, and terminally differentiated cells are absent. With the increase of the applied strain this proportion changes with the fraction of original stem cells quickly diminishing to zero. The terminally differentiated cells appear at about a 3% strain, and ultimately they become the majority and comprise about 80% of the total population. Finally, Fig 5B shows the strain dependence of the time where n_o_ reaches the maximum over the interval of 21 days (thin line) and the time when n_4_ starts deviating from zero. For smaller strains (below 2%), n_0_ is maximal at the end point of the interval (day 21). Then, for larger strains, when n_o_ starts decreasing after strain application, this time switched to day 3. For strains below 4%, the terminally differentiated cells do not appear by day 21. When a strain of about 4% is applied, the terminally differentiated cells start appearing at an earlier time point, which drops to day 10 for the strain of 15%.

## Model and Computational Method

The schematic of our approach is shown in Fig 1, and in this section, we describe the main components of our model and computational details of its implementation. First, we present the system of ODEs for the kinetics of ASC myogenesis, and then, we show how we incorporate the strain dependence into the model. After this, we discuss the computation of the conditions associated with the interaction of the cell with its environment. Then, we describe the computational implementation of the feedback factor due to the limit on cell density. Finally, we discuss the particular behavior of the stage#1 (cell expressing the factor PAX7) in our model.

**Fig 6:**
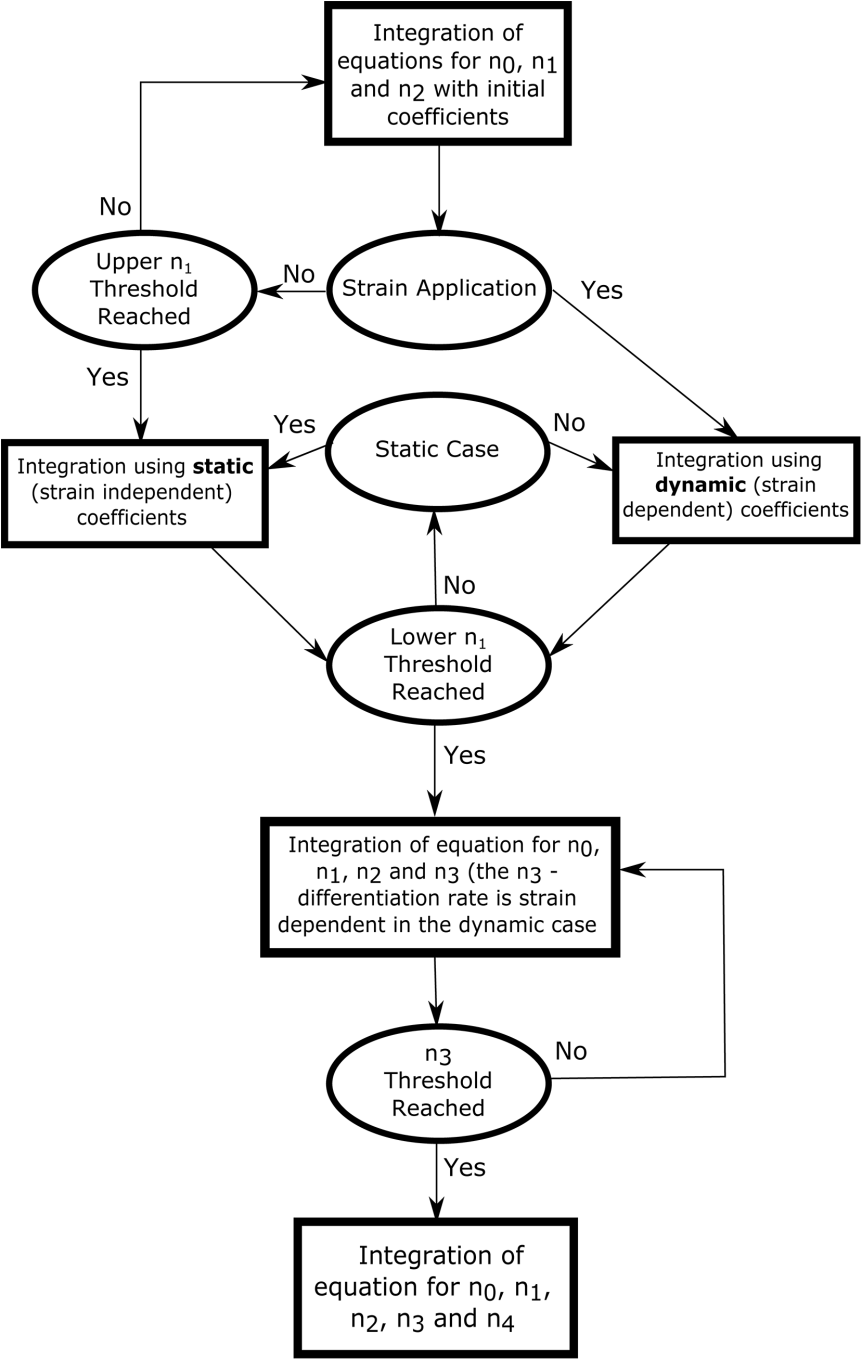
Flow chart of the computational method with switches between versions of the kinetic equations. The flow chart shows the conditions that must be met before integrating the corresponding versions of kinetic equations. The figure additionally illustrates the underlying logic of the computation.

A flow chart depicting our computational method is presented in Fig 6.

### Kinetic Equations

As previously mentioned, our modeling approach is implemented as a system of ODEs whose parameters change at certain moments of time but are constants in between those moments. The stage-to-stage transitions and parameter switching are not fixed in advance but rather obey conditions associated with cell/ECM or cell-cell signaling and are hence expressed in terms of the system variables. Furthermore, the rate of the growth of cell numbers in different stages is constrained due to a physical limit of cell density in the culture, which is implemented via a feedback function of the total cell number. These features make the problem nonlinear, as opposed to its linear counterpart presented in [8].

The equations describing the kinetics of ASC myogenesis are derived as conditions of the flux balance for each of the five stages at a given moment of time. These equations take the form

$$\frac{dn_{0}}{dt}=\left[\left(2r_{0}-1\right)p_{0}n_{0}-d_{0}n_{0}\right]f\left(n_{tot}\right)$$ (7)

$$\frac{dn_{i}}{dt}=\left[\left(2r_{i}-1\right)p_{i}n_{i}-d_{i}n_{i}+2\left(1-r_{i-1}\right)p_{i-1}n_{i-1}\right]f(n_{tot})\;i=1,2$$ (8)

$$\frac{dn_{3}}{dt}=[p_{3}n_{3}-d_{3}n_{3}+2\left(1-r_{2}\right)p_{2}n_{2}-Dn_{3}]f\left(n_{tot}\right)$$ (9)

$$\frac{dn_{4}}{dt}=\left[Dn_{3}-d_{4}n_{4}\right]f\left(n_{tot}\right)$$ (10)

The first terms in the square brackets on the right-hand sides of Eqs 7–9 correspond to the number of self-renewed cells associated with the proliferation process. The second terms in the same brackets correspond to the cell removal due to their death. The third terms in the square brackets in Eqs 8 and 9 correspond to the influx of cells differentiated in the previous stage via cell asymmetric division. The fourth term in the square brackets in Eq 9 and the first terms in Eq 10, respectively, correspond to the direct differentiation from the pre-final to final stage of ASC myogenesis. Finally, the function after the square brackets in Eqs 7–10 corresponds to the feedback factor due to the limit in the cell density.

### Strain Dependence

The coefficients in Eqs 7–10 are strain-dependent, and they are assumed to be linear functions of based on the previously determined values for 10% and 0% strain. The values for the parameters corresponding to the 10% and 0% strain as presented above in Table 1 are estimated by matching the experimental data in the dynamic and static cases, respectively. Thus, the coefficients k_j_ (r_0_, p_0_, d_0_, r_1_, p_1_, d_1_, r_2_, p_2_, d_2_, r_3_, p_3_, d_3_, D_3_, and d_4_) of the system (7–10) are approximated by strain-linear functions as follows

$$k_{j}(\varepsilon )=k_{j}^{st}+(\frac{k_{j}^{dyn}-k_{j}^{st}}{0.10})(\varepsilon )$$ (11)

where k_j_^st^ and k_j_^dyn^ are the values of the corresponding parameter under static and dynamic conditions and ε is the applied (dimensionless) strain expressed as a decimal. This approximation automatically determines the strain dependence of the parameter combinations, *l*_i_, (Eqs 9 and 10) that are key to the pattern of the kinetics of ASC myogenesis. In addition, the conditions that determine the switches between different sets of ODEs are conceptually the same for all strains; however, the actual moments of such switches (except the first of them due to the strain application on day 3) are strain-dependent and they change with the approximation delineated by Eq 11. While the strain approximation of the model rates is monotonic and linear, the resulting kinetics exhibit qualitative changes in the pattern of the system behavior, such as switches from increasing to decreasing in the number of stem cells (Fig 4), switches in moments (days) of reaching the maximal fraction of the stem cell (Fig 5A), switches in moments (days) of appearance of the terminally differentiated cells (Fig 5B), etc. Finally, the linear strain-dependence used here is for a limited range of strain. It is exactly dynamic at 10% and applied up to 15%. Its nonlinear correction as well as additional experimental testing is beyond the scope of the current paper but will be done in the future studies.

### Smooth Switches

Step-wise switches in the ODE system parameters may cause the overall solution to have slope discontinuities. In order to avoid this, a smoothening technique is applied to provide a more gradual change in the system parameters. This technique is implemented in four cases associated with strain-application (dynamic case), reaching the n_1_-treshold (static and dynamic cases), n_3_-release (static and dynamic cases), and n_4_-release (dynamic case). The first three of such cases are treated by introducing a gradual change in the parameter combination *l*_*i*_ (Eq 5). The last case is treated by using a gradually changing direct differentiation rate, D. Below, we present our analytical approach in the case where the l_1_-parameter changes from a positive value *l* ^*+*^ to a negative value *l* ^*-*^ near the moment of strain application (the two other cases associated with a change in parameter *l*_*i*_ are treated similarly)

$$l_{1}=\{\begin{array}{c} l^{+}\;n_{1}\leq n_{1}^{*}\\ l\left(n_{1}-n_{1}^{*}\right)+l^{+}\;n_{1}^{*}<n_{1}\leq n_{1}^{\mathrm{**}}\left(\frac{dn_{1}}{dt}\geq 0\right)\\ \left(l+\frac{l^{+}-l^{-}}{n_{1}^{\mathrm{**}}-n_{1}^{*}}\right)\left(n_{1}-n_{1}^{*}\right)+l^{+}\;n_{1}^{*}\leq n_{1}<n_{1}^{\mathrm{**}}\;(\frac{dn_{1}}{dt}<0)\\ l^{-}\;n_{1}<n_{1}^{*} \end{array}$$ (12)

**Fig 7:**
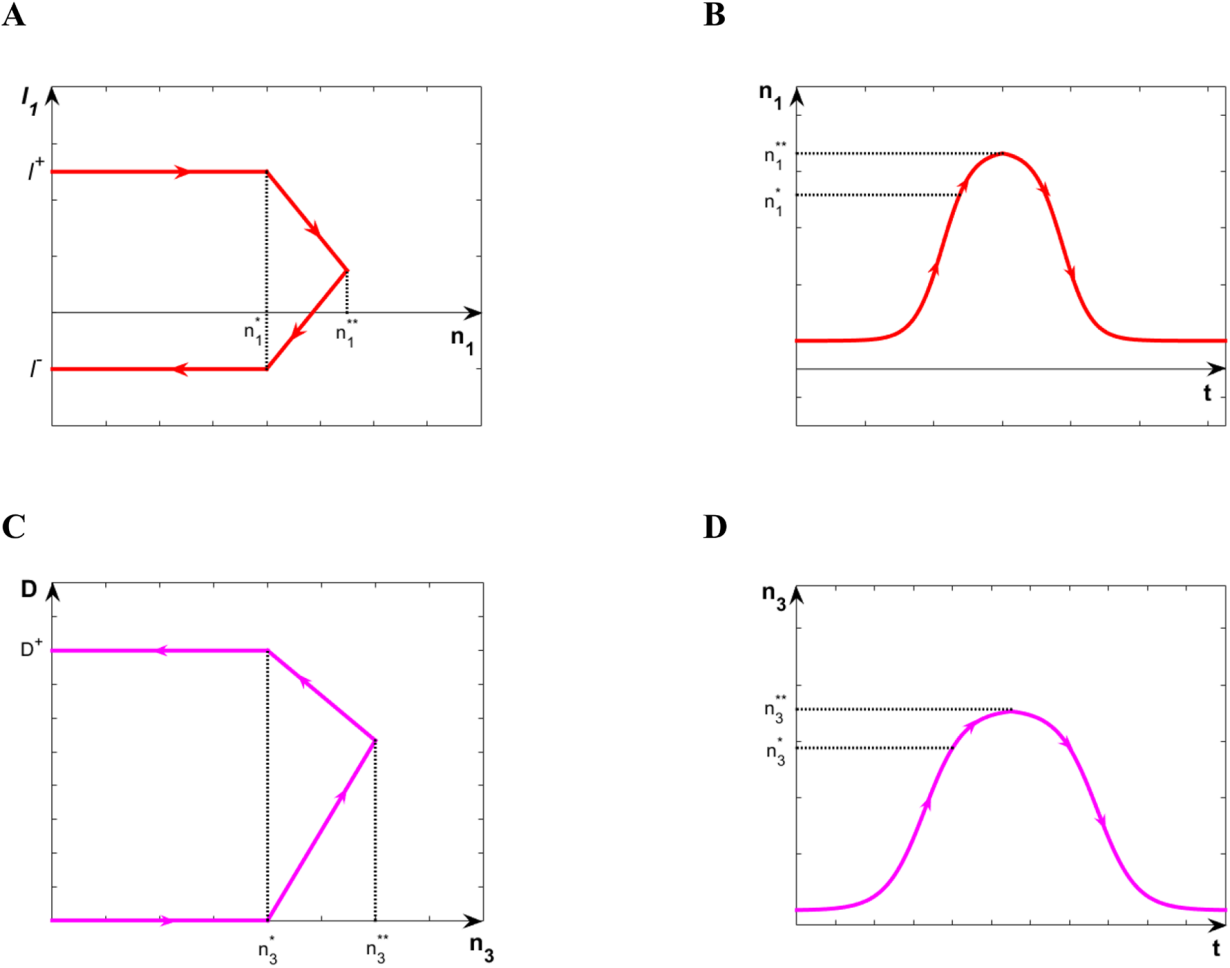
Illustration of the smoothening technique. **(**A) Evolution of *l*_*1*_-composite parameter vs. n_1_ and (B) corresponding evolution of n_1_ vs. time near n_1_* threshold. (C) Evolution of the direct differentiation rate, D, vs. n3 and (D) corresponding evolution of n_3_ vs, time near n_3_* threshold.

The kinetic equation for n_1_ is integrated by values of *l* that depend on what the value of n_1_ is. As shown by Eq 12, the progression of *l*_*1*_ can be described as a piecewise function with four components. Initially, while n_1_ is below n_1_^*^ (a chosen threshold), “the static” value, *l*_*1*_ = *l* ^*+*^, is used during which n_1_ increases. When n_1_ reaches n_1_^*^, *l*_*1*_ begins to linearly decrease as n_1_ continues to increase. This behavior continues until n_1_** is reached. At n_1_**, dn_1_/dt = 0. Moreover, n_1_**is slightly above n_1_* (1.05–1.1 x n_1_*). Such a relationship between n_1_* and n_1_** is provided by the choice of the coefficient *l* in Eq 12. After n_1_**, *l*_*1*_ linearly decreases with a different slope, and n_1_ decreases during this stretch as well. After reaching the point where n_1_ = n_1_*again, the kinetic equation for n_1_ is integrated with the “dynamic” value of *l*_*1*_ = *l* ^*-*^, and n_1_ continues to decrease. The sketch of the evolution of *l*_*1*_ and the corresponding behavior of n_1_ is shown in Fig 7A and 7B.

In the case of the direct differentiation during the transition of cells from stage#3 to #4, the analytical expression for the gradually changing differentiation rate, D, takes the following form:

$$D=\{\begin{array}{c} 0\;n_{3}\leq n_{3}^{*}\\ k\left(n_{3}-n_{3}^{*}\right)\;n_{3}^{*}<n_{3}\leq n_{3}^{\mathrm{**}}\left(\frac{dn_{3}}{dt}\geq 0\right)\\ \left(k-\frac{D^{+}}{n_{3}^{\mathrm{**}}-n_{3}^{*}}\right)n_{3}+\frac{D^{+}n_{3}^{\mathrm{**}}}{n_{3}^{\mathrm{**}}-n_{3}^{*}}-kn_{3}^{*}\;n_{3}^{*}\leq n_{3}<n_{3}^{\mathrm{**}}\;(\frac{dn_{3}}{dt}<0)\\ D^{+}\;n_{3}<n_{3}^{*} \end{array}$$ (13)

Once again, the values of D can be described by a piecewise function also with four compartments. Indeed, Eq 13 describes the gradual change of the differentiation rate from 0 (before the n_4_—release) to a positive value, D^+^ (after the direct differentiation starts). A notation consistent with that of Eq 12 has been adopted in the formulation of Eq 13. The changes in the rate D and the corresponding evolution of n_3_ are sketched in Fig 7C and 7D.

### The Feedback Factor

The feedback factor, f(n_tot_) is a function of the total cell number that does not considerably alter cell kinetics while the total cell number is significantly below the threshold, n^‡^. The usage of such a factor prevents unhindered exponential growth, allowing it to better model biological systems. A feedback factor of the following form was chosen to be implemented

$$f=1/\left[1+e^{s\left(n_{tot}-n^{‡}\right)}\right]$$ (14)

As the cell count approaches the threshold, however, the feedback factor causes the rate of increase of cell numbers to decrease across all stages. In addition, as soon as the total cell number is slightly above n^‡^, the magnitude of the factor drops to value that is practically zero; in turn, the changes in cell numbers are stabilized for the corresponding stages. In addition, the threshold, n^‡^ is also the value that corresponds to a 50% drop in the rate of cell number increase in all five stages. A computational parameter, *s*, modulates how quickly the rate of cell number increase drops to zero after the threshold cell count is met. In the model, the values of n^‡^ ≈ 3–3.5x10^5^ and s ≈ 3-4x10^-5^ were used. The biological analogue of such a factor can be the result of signals from a protein transiently expressed when the total cell number approaches n^‡^ but whose effects persist throughout the myogenic process. The feedback factor presented above is similar to functions used to describe inhibitory-signal processes [17, 19].

### Stage 1 Suppression

As referenced before, studies have found a mutual inhibition between the PAX7 and MyoD markers [21, 22]. Although this biological factor has been incorporated into the model, it was initially found that the n_1_ count increases even with MyoD expression in the static and quasi-static cases. It was determined that this can be attributed to the aforementioned feedback factor that provides long-term stabilization. Thus, in order to accurately represent the inhibition, another threshold for was imposed on n_1_, where dn_1_/dt = 0 for any n_1_ above the threshold. This was not observed in the dynamic case, and consequently, this additional threshold did not have any effect in the dynamic case and quasi-dynamic cases.

### Future Development of the Model

The proposed model describes a fine time-dependent multistage picture of ACS myogenesis, but it assumes a complete spatial homogeneity throughout the area occupied by the cells. To take into account the gradients of the external factors, 3-D geometry, etc., the kinetic equations used here can be incorporated into a PDE model that also accounts for spatial coordinates and the corresponding terms describing the dependence of myogenic processes on these coordinates as it was successfully done in prior cell studies [31]. Alternatively, the concepts of the current method can used for time-discrete rule-based models to compute the time evolving spatial distribution of cells in different stages of differentiation [32]. Another direction of the model development can be a more detailed characterization of cell/ECM and cell/cell signaling obtained based on additional model-stimulated experiments. Finally, a more advanced model can be used to further optimize the experimental procedures, including the most informative set of the reported proteins, time and frequency of the strain application, etc.

## References

[1] Zuk PA, Zhu M, Mizuno H, Huang J, Futrell JW, Katz AJ, et al Multilineage cells from human adipose tissue: implications for cell-based therapies. Tissue Eng. 2001 4; 7(2): 211–228. 11304456 10.1089/107632701300062859

[2] Zuk PA, Zhu M, Ashjian P, DeUgarte DA, Huang JI, Mizuno H, et al Human adipose tissue is a source of multipotent stem cells. Mol Biol Cell. 2002 12; 13(12): 4279–4295. 12475952 10.1091/mbc.E02-02-0105 PMC138633

[3] Hutton DL, Logsdon EA, Moore EM, Mac Gabhann F, Gimble JM, Grayson WL. Vascular morphogenesis of adipose-derived stem cells is mediated by heterotypic cell-cell interactions. Tissue Eng Part A. 2012 8; 18(15–16): 1729–1740. 10.1089/ten.TEA.2011.0599 22462659 PMC3419853

[4] Hutton DL, Moore EM, Gimble J, Grayson WL. Platelet-derived growth factor and spatiotemporal cues induce development of vascularized bone tissue by adipose-derived stem cells. Tissue Eng Part A. 2013 9; 19(17–18): 2076–2086. 10.1089/ten.TEA.2012.0752 23582144 PMC3725877

[5] Wu L, Cai X, Zhang S, Karperien M, Lin Y. Regeneration of articular cartilage by adipose tissue derived mesenchymal stem cells: perspectives from stem cell biology and molecular medicine. J Cell Physiol. 2013 5; 228(5): 938–944. 10.1002/jcp.24255 23042088

[6] Ashjian PH, Elbarbary AS, Edmonds B, DeUgarte D, Zhu M, Zuk PA, et al In vitro differentiation of human processed lipoaspirate cells into early neural progenitors. Plast Reconstr Surg. 2003 5; 111(6): 1922–1931. 12711954 10.1097/01.PRS.0000055043.62589.05

[7] Mizuno H, Zuk PA, Zhu M, Lorenz HP, Benhaim P, Hedrick MH. Myogenic differentiation by human processed lipoaspirate cells. Plast Reconstr Surg. 2002 1; 109(1): 199–209; discussion 210–211. 11786812 10.1097/00006534-200201000-00030

[8] Huri PY, Wang A, Spector AS, Grayson WL. Multistage adipose-derived stem cell myogenesis: an experimental and modeling study. Cell Mol Bioeng. 2014 12; 7(4): 497–509. 10.1007/s12195-014-0362-7

[9] Huri PY, Cook CA, Hutton DL, Goh BC, Gimble JM, DiGirolamo DJ, et al Biophysical cues enhance myogenesis of human adipose derived stem/stromal cells. Biochem Biophys Res Commun. 2013 8; 438(1): 180–185. 10.1016/j.bbrc.2013.07.049 23876311 PMC3960302

[10] McBeath R, Pirone DM, Nelson CM, Bhadriraju K, Chen CS. Cell shape, cytoskeletal tension, and RhoA regulate stem cell lineage commitment. Dev Cell. 2004 4; 6(4): 483–495. 15068789 10.1016/s1534-5807(04)00075-9

[11] Engler AJ, Sen S, Sweeney HL, Discher DE. Matrix elasticity direct stem cell lineage specification. Cell. 2006 8; 126(4): 677–689. 16923388 10.1016/j.cell.2006.06.044

[12] Ankam S, Suryana M, Chan LY, Moe AA, Teo BK, Law JB, et al Substrate topography and size determine the fate of human embryonic stem cells to neuronal or glial lineage. Acta Biomater. 2013 1; 9(1): 4535–4545. 10.1016/j.actbio.2012.08.018 22906625

[13] Dalby MJ, Gadegaard N, Oreffo RO. Harnessing nanotopography and integrin-matrix interactions to influence stem cell fate. Nat Mater. 2014 6; 13(6): 558–569. 10.1038/nmat3980 24845995

[14] Lee WC, Maul TM, Vorp DA, Rubin JP, Marra KG. Effects of uniaxial cyclic strain on adipose-derived stem cell morphology, proliferation, and differentiation. Biomech Model Mechanobiol. 2007 7; 6(4): 265–273. 16906436 10.1007/s10237-006-0053-y

[15] Maul TM, Chew DW, Nieponice A, Vorp DA. Mechanical stimuli differentially control stem cell behavior: morphology, proliferation, and differentiation. Biomech Model Mechanobiol. 2011 12; 10(6): 939–953. 10.1007/s10237-010-0285-8 21253809 PMC3208754

[16] Huang S, Guo YP, May G, Enver T. Bifurcation dynamics in lineage-commitment in bipotent progenitor cells. Dev Biol. 2007 5; 305(2): 695–713. 17412320 10.1016/j.ydbio.2007.02.036

[17] Alon U. An introduction to systems biology: Design principles of biological circuits 1st ed. Chapman & Hall/CRC Press; 2006.

[18] Zandstra PW, Lauffenburger DA, Eaves CJ. A ligand-receptor signaling threshold model of stem cell differentiation control: a biologically conserved mechanism applicable to hematopoiesis. Blood. 2000 8; 96(4): 1215–1222. 10942360

[19] Marciniak-Czochra A, Stiehl T, Ho AD, Jager W, Wagner W. Modeling of asymmetric cell division in hematopoietic stem cells—regulation of self-renewal is essential for efficient repopulation. Stem Cells Dev. 2009 4; 18(3): 377–385. 10.1089/scd.2008.0143 18752377

[20] Agur Z, Kirnasovsky OU, Vasserman G, Tencer-Hershkowicz L, Kogan Y, Harrison H, et al Dickkopf1 regulates fate decision and drives breast cancer stem cells to differentiation: an experimentally supported mathematical model. PloS One. 2011; 6(9): e24225 10.1371/journal.pone.0024225 21915302 PMC3167819

[21] Olguin HC, Yang Z, Tapscott SJ, Olwin BB. Reciprocal inhibition between Pax7 and muscle regulatory factors modulates myogenic cell fate determination. J Cell Biol. 2007 6; 177(5): 769–779. 17548510 10.1083/jcb.200608122 PMC2064278

[22] Halevy O, Piestun Y, Allouh MZ, Rosser BW, Rinkevich Y, Reshef R, et al Pattern of Pax7 expression during myogenesis in the posthatch chicken establishes a model for satellite cell differentiation and renewal. Dev Dyn. 2004 11; 231(3): 489–502. 15390217 10.1002/dvdy.20151

[23] Chargé SB, Rudnicki MA. Cellular and molecular regulation of muscle regeneration. Physiol Rev. 2004 1; 84(1): 209–238. 14715915 10.1152/physrev.00019.2003

[24] Le Grand F, Rudnicki MA. Skeletal muscle satellite cells and adult myogenesis. Curr Opin Cell Biol. 2007 12; 19(6): 628–633. 17996437 10.1016/j.ceb.2007.09.012 PMC2215059

[25] Zaidel-Bar R, Cohen M, Addadi L, Geiger B. Hierarchical assembly of cell-matrix adhesion complexes. Biochem Soc Trans. 2004 6; 32(Pt3): 416–420. 15157150 10.1042/BST0320416

[26] Zamir E, Geiger B. Molecular complexity and dynamics of cell-matrix adhesions. J Cell Sci. 2001 10; 114(Pt 20): 3583–3590. 11707510 10.1242/jcs.114.20.3583

[27] Quach NL, Biressi S, Reichardt LF, Keller C, Rando TA. Focal adhesion kinase signaling regulates the expression of caveolin 3 and beta1 integrin, genes essential for normal myoblast fusion. Mol Biol Cell. 2009 7; 20(14): 3422–3435. 10.1091/mbc.E09-02-0175 19458188 PMC2710835

[28] Doherty JT, Lenhart KC, Cameron MV, Mack CP, Conlon FL, Talyor JM. Skeletal muscle differentiation and fusion are regulated by the BAR-containing Rho-GTPase-activating protein (Rho-GAP), GRAF1. J Biol Chem. 2011 7; 286(29): 25903–25921. 10.1074/jbc.M111.243030 21622574 PMC3138304

[29] Charrasse S, Comunale F, Grumbach Y, Poulat F, Blangy A, Gauthier-Rouviére C. RhoA GTPase regulates M-cadherin activity and myoblast fusion. Mol Biol Cell. 2006 2; 17(2): 749–759. 16291866 10.1091/mbc.E05-04-0284 PMC1356585

[30] Duan R, Jin P, Luo F, Zhang G, Anderson N, Chen EH. Group I PAKs function downstream of Rac to promote podosome invasion during myoblast fusion in vivo. J Cell Biol. 2012 10; 199(1): 169–185. 10.1083/jcb.201204065 23007650 PMC3461515

[31] Osborne JM, Walter A, Kershaw SK, Mirams GR, Fletcher AG, Pathmanathan P, et al A hybrid approach to multi-scale modelling of cancer. Philos Trans A Math Phys Eng Sci. 2010 11; 368(1930): 5013–5028. 10.1098/rsta.2010.0173 20921009

[32] Hwang M, Garbey M, Barceli SA, Tran-Son-Tay R. Rule-based simulation of multi-cellular biological systems–a review of modeling techniques. Cell Mol Bioeng. 2009 9; 2(3): 285–294. 21369345 10.1007/s12195-009-0078-2 PMC3045734

